# Application of atomic force microscopy in cancer research

**DOI:** 10.1186/s12951-018-0428-0

**Published:** 2018-12-11

**Authors:** Xiangying Deng, Fang Xiong, Xiayu Li, Bo Xiang, Zheng Li, Xu Wu, Can Guo, Xiaoling Li, Yong Li, Guiyuan Li, Wei Xiong, Zhaoyang Zeng

**Affiliations:** 1 The Key Laboratory of Carcinogenesis of the Chinese Ministry of Health and Hunan Key Laboratory of Translational Radiation Oncology, Hunan Cancer Hospital and The Affiliated Cancer Hospital of Xiangya School of Medicine, Central South University, Changsha, 410013 Hunan China; 2 The Key Laboratory of Carcinogenesis and Cancer Invasion of the Chinese Ministry of Education, Cancer Research Institute and School of Basic Medical Science, Xiangya School of Medicine, Central South University, Changsha, 410078 China; 3 Hunan Key Laboratory of Nonresolving Inflammation and Cancer, Disease Genome Research Center, The Third Xiangya Hospital, Central South University, Changsha, 410013 Hunan China; 4 Department of Chemistry, University of North Dakota, Grand Forks, ND 58202 USA; 5 Department of Cancer Biology, Lerner Research Institute, Cleveland Clinic, Cleveland, OH 44195 USA

**Keywords:** Atomic force microscopy, Cancer cells, Morphology, Mechanical properties

## Abstract

Atomic force microscopy (AFM) allows for nanometer-scale investigation of cells and molecules. Recent advances have enabled its application in cancer research and diagnosis. The physicochemical properties of live cells undergo changes when their physiological conditions are altered. These physicochemical properties can therefore reflect complex physiological processes occurring in cells. When cells are in the process of carcinogenesis and stimulated by external stimuli, their morphology, elasticity, and adhesion properties may change. AFM can perform surface imaging and ultrastructural observation of live cells with atomic resolution under near-physiological conditions, collecting force spectroscopy information which allows for the study of the mechanical properties of cells. For this reason, AFM has potential to be used as a tool for high resolution research into the ultrastructure and mechanical properties of tumor cells. This review describes the working principle, working mode, and technical points of atomic force microscopy, and reviews the applications and prospects of atomic force microscopy in cancer research.

## Background

As unconstrained and rapidly dividing cells, the physicochemical properties of cancer cells have changed in comparison with the normal cells from which they are derived [1]. During the invasion and metastasis of cancer cells, the adhesion between cells is reduced, and the shape and hardness of the cells changes according to the surrounding environment [2–4], in order to meet the physiological activities of cancer cells themselves. Therefore, researchers can determine whether or not cells are cancerous, whether the cancer cells are invasive or metastasize, and the effects of drugs on cancer cells and so on by the physical properties such as hardness, adhesion, and Young’s modulus. However, in order to achieve the above research, it is necessary to observe and manipulate cells at a nanometer level of resolution. Atomic force microscopy (AFM) is an extremely high resolution tool, which can be used to observe the morphology of a sample, and quantitatively measure its mechanical properties at atomic resolution. For this reason, AFM has applications in cancer research. The technique was invented in 1986 by IBM’s G. Binnig, and C. F. Quate and C. Gerber at Stanford University [5]. AFM is the highest-resolution and most widely used member of the scanning force microscopy (SFM) family, with a horizontal resolution of 0.1 nm, a vertical resolution of 0.01 nm, and an atomic level of resolution. It goes beyond the limits of the resolution of microscopes which use light and electron wavelengths. AFM uses a microscopic physical probe to “grope” the microcosm and observes the morphology of the sample under investigation in three-dimensional space, obtaining information from the very weak interaction between the probe and the sample surface.

The technical advantages of AFM are the main reasons for its rapid and widespread adoption in biology and medicine [6]. Firstly, due to the extremely high resolution of AFM, it is possible to perform direct three-dimensional imaging of molecular, and even atomic-scale structures. Secondly, sample preparation for AFM is straightforward, the damage to the original structure is small, and the original appearance of the sample can be determined objectively and accurately. Thirdly, due to the fact that samples can be observed under near-physiological conditions, the dynamic processes of molecules, organelles, and other structures in living cells can be recorded in real time by AFM [7]. Fourthly, AFM can measure intermolecular forces, charge, pH, and other physicochemical characteristics of sample materials. Finally, the functionalized probe can be used to identify specific molecules or interaction forces such as ligand-receptor interactions. Therefore, AFM has good prospects for application in biomedicine and clinical medicine, and particularly in the diagnosis and treatment of cancer. Cell mechanics is a promising biomarker for indicating cell states [8–13]. The mechanics of cells can be measured by many methods including, among others, magnetic twisting cytometry, optical tweezers, and atomic force microscopy. Of these methods, however, AFM is the most widely used tool for this purpose [14–16]. The advantages and disadvantages of these methods are summarized in Table 1.

**Table 1:** Comparison of representative methods in measuring cell mechanics [8, 14–16]

Methods	Advantages	Disadvantages
Microfluidics	High throughput (~ 1 cell/s); ability to control cell environment and approximate physiological conditions	Be prone to cell adhesion and clogging; limited materials for fabricating devices; cell size is often neglected
Micropipette aspiration	Simple and cost-effective; large range of force (up to ~ 100 nN)	Low throughput; limited special resolution (< 1 cell/10 min); possible damage to cells; mainly for suspended cells
Micropost arrays	Ability to measure the traction forces of single cells or cell populations	Mainly for adherent cells; high cost and complexity; the topology of micropost arrays may influence cell activities
Magnetic twisting cytometry	Probing the local mechanics of cells; magnetic beads can be bound with diverse types of cellular molecules or structures	Low throughput (< 1 cell/min); difficult to standardize; only for unidirectional forces
Optical tweezers	High precision measurements of small forces (0.01–10^3^ pN); can be integrated with microfluidic delivery	Limited force (< 500 pN) applied on cells; detrimental effects on cells due to heating
Parallel plate	Simple and cost-effective; ability to study single cells of cell populations	Low throughout; low spatial resolution
Atomic force microscopy	Applicable for both suspended cells and adherent cells; simultaneously obtain structural and mechanical information with nanometer resolution	Low throughput (< 1 cell/10 min); the mechanical poking of the AFM tip may influence cell activities

At present, the study of the physical properties of tumors is not sufficiently thorough. In view of the various unique advantages of AFM, it may be used to investigate changes in ultrastructure and mechanical properties within tumor tissues and cells which, in turn, can be used as a basis for clinical adjuvant diagnosis [17]. At the same time, AFM allows for the exploration of the mechanisms of antitumor drugs at the cellular and molecular level; this allows for the evaluation of drug efficacy, and opens avenues for the prevention of tumor cell proliferation [18–22].

## The basic principle of AFM

**Fig. 1:**
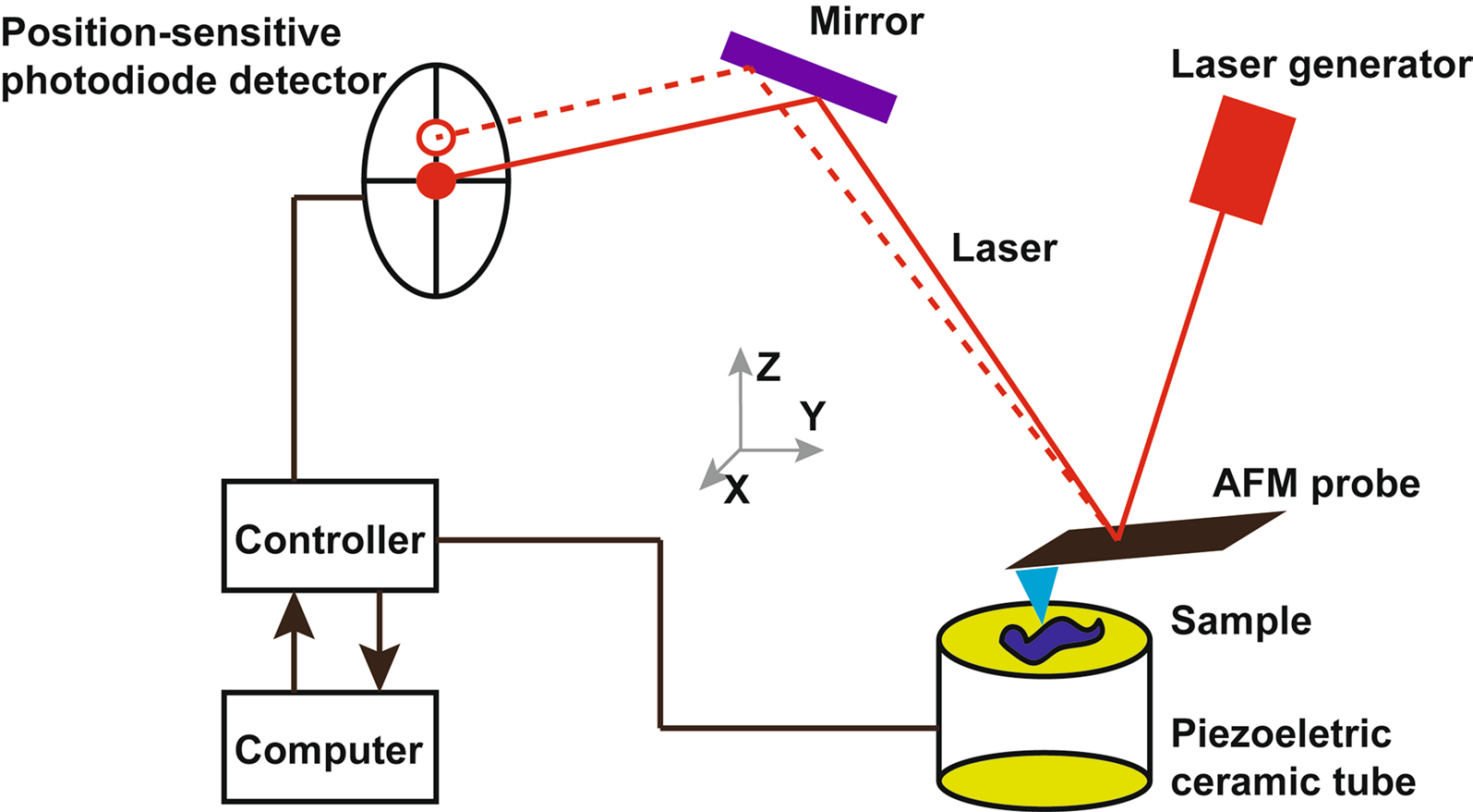
Schematic diagram of AFM working principles. The AFM instrument is composed of a piezoelectric ceramic tube, a laser generator, a position-sensitive photodiode detector, a controller, and an AFM probe. The AFM probe is a micro-cantilever with a sharp tip attached at its end. The tip, which has a monomolecular point, allows for nanometer resolution imaging and the micro-cantilever is a force sensor that can detect even minute deformation of a sample, enabling very high sensitivity AFM in force measurements

**Fig. 2:**
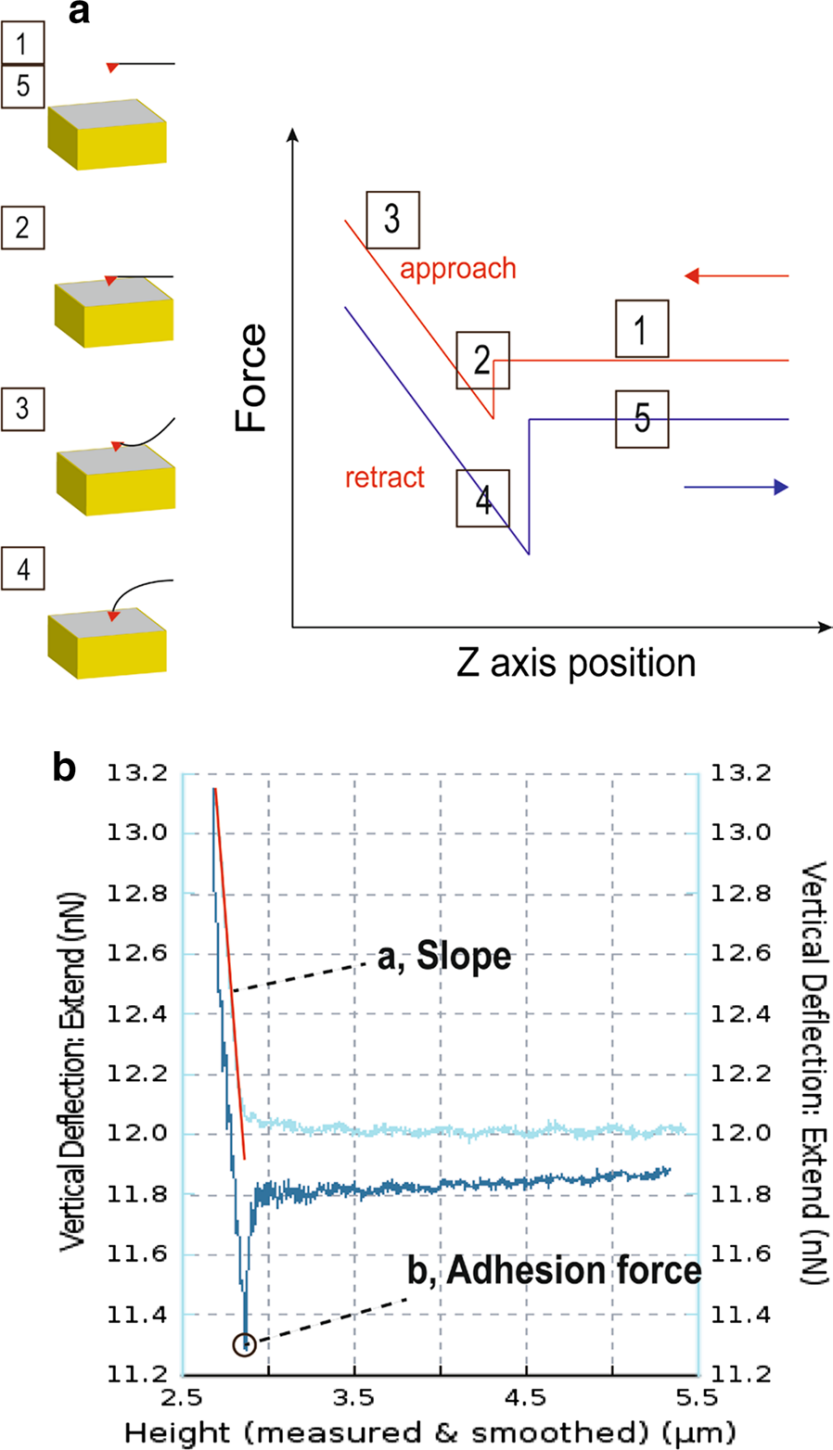
Force-distance curve-based AFM. **a** Principle of force-distance (FD) curves by approaching (red) and withdrawing (blue) the AFM tip from the sample. The tip of the cantilever is initially distant from the sample [1] to which it is brought into contact [2]. During retraction [3] of the AFM tip, adhesive events may occur at different distances due to nonspecific [4] or specific [5] interactions between tip and sample. **b** The force-curve plot from an AFM measurement. The slope value is fitted with a linear fit (red line) (**a**), the adhesion is measured as a single value and the mechanical value of the point (**b**) indicates the adhesion force

The AFM system consists of the following components: a micro-cantilever with probe, a micro-cantilever motion detection device, a feedback loop for monitoring micro-cantilever motion, a piezoelectric ceramic scanning device for sample scanning, and a computer-controlled image acquisition, display, and processing system (Fig. 1). AFM studies the surface structure and properties of the sample by detecting very weak interatomic interactions between the sample surface and the probe tip. The working principle is to fix one end of micro cantilever, which is extremely sensitive to weak force, while other end of cantilever contained in the probe brought into close proximity with the sample [23]. Once this has been accomplished, a very weak force, which may be either repulsive or attractive, exists between the tip atom of the probe and the atoms of the sample surface. The magnitude of this force changes the deformation of the micro cantilever or its motion state. When the sample is scanned, sensors are used to detect these changes and force distribution information is obtained, allowing the user to obtain surface structure information with nanometer resolution. The AFM scanner can move in the X, Y, and Z directions. While the distance traveled in the X and Y directions varies with the scanner, the vertical Z direction is typically limited to a few microns. At the same time, the morphology of the scanning area sample can be obtained by reconstructing the position of the piezoelectric ceramic scanning tube in the Z-direction and the X–Y plane. The Young’s modulus value of samples, like cells and tissues, is fitted with a linear fit by Hertz model, with a smaller Young’s modulus indicating that the sample is more susceptible to deformation. The force-distance curve measured by AFM reflects the quantitative force between the tip and the sample. Using the corresponding probes, equipped with dedicated software, AFM can measure the nano-indentation of the micro regions, and force–displacement curves drawn on this basis [24] can be used for the quantitative measurement of a material’s elastic modulus, adhesion, and stiffness (Fig. 2) [25]. Each force curve is saved during the test for later quantitative analysis.

**Fig. 3:**
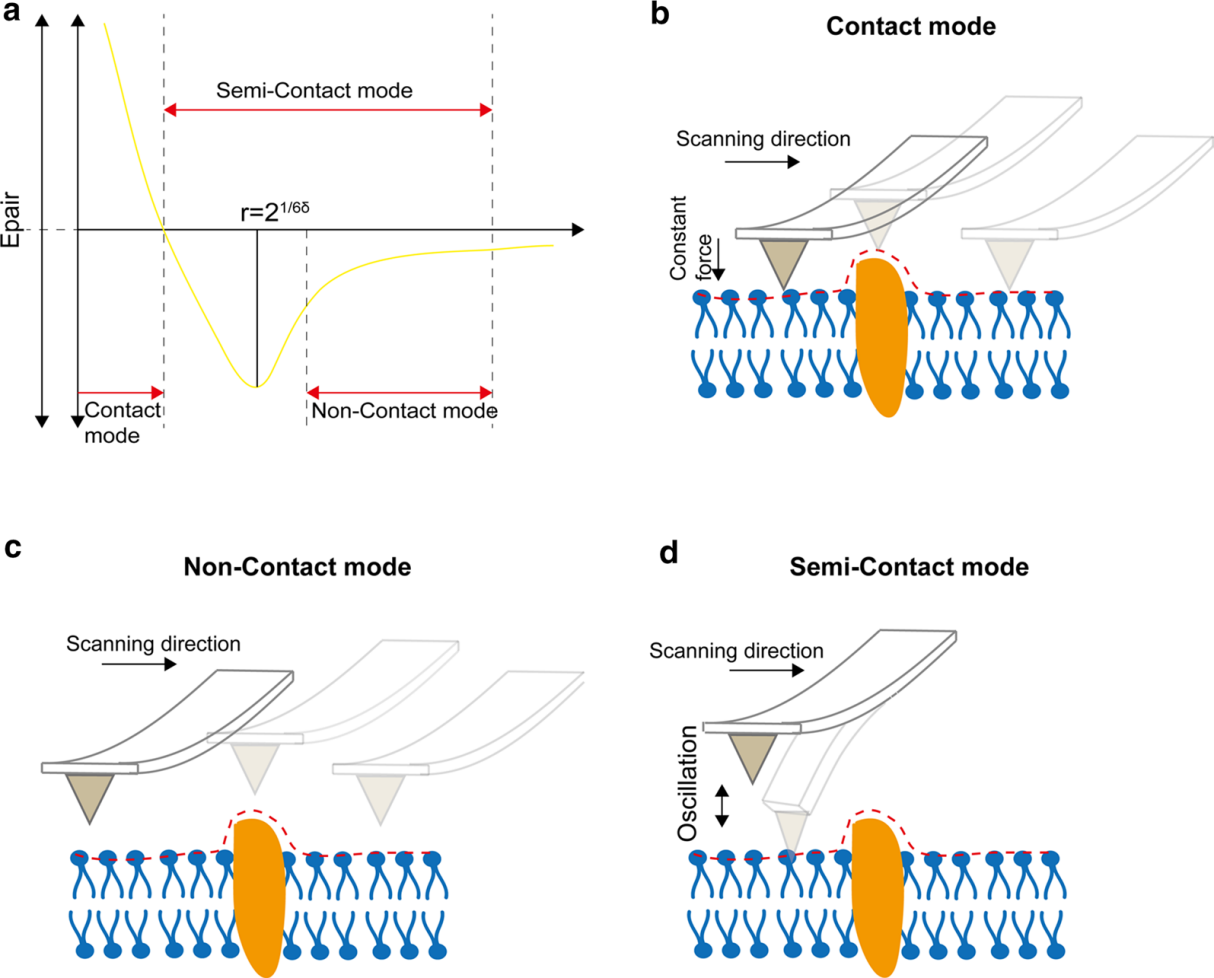
Three basic working modes of AFM. **a** The curve of both interatomic force and intervals relation. **b** In contact mode the probe is always slightly contact with the sample and scanned in a constant force mode. **c** In the non-contact mode the tip of the needle always vibrates on the surface of the sample, but it is never contact with the sample. The scanning detector detects long-range forces such as van der Waals force or electrostatic forces that do not damage the imaged sample. **d** In the tapping mode the micro cantilever is subjected to stress vibration near its resonant frequency, and the oscillating needle tip gently strikes the surface of the sample, intermittently making contact with it

The principle of AFM imaging and mechanical property detection is based on the attraction and repulsive forces between atoms. The atoms at the tip of the probe and those on the surface of the sample are subjected to an attractive force when too far apart, and to a repulsive force when they are in close proximity (Fig. 3a). The basic imaging modes of AFM include: contact mode (Fig. 3b), non-contact mode (Fig. 3c), and tapping or semi-contact mode (Fig. 3d). In contact mode, the probe of AFM keeps a slight contact with the sample surface and maintains a constant force. In non-contact mode, the surface topography of the sample is produced by measuring the atomic attraction between the probe and the sample. In the tapping mode, the micro-cantilever of the probe is forced to move near the resonance frequency, and the probe makes contact with the sample intermittently. The force between the probes and sample can be kept constant by controlling the amplitude or deflection of the micro-cantilever when the probe touches the sample. Tapping mode effectively eliminates the influence of the lateral force and reduces the force caused by the adsorption layer, resulting in a high image resolution, especially for surface ultrastructure observation of biological samples.

The study of single cancer cells using AFM is expected to be advantageous in two aspects. On the one hand, the diagnosis of early cancer focuses on the observation of changes and differences between single cells, with the differences between cancerous and non-cancerous cells allowing for early diagnosis and treatment of cancer [26]. On the other hand, it allows for the study of the structure and function of cancer cells, the mechanisms involved in their spreading, interaction processes between cells, and the mechanisms of anti-cancer drugs, etc. The resultant findings from such studies should aid researchers in their goals of finding means of blocking cancer cell proliferation, and developing anti-cancer drugs [27–30].

## Observation of cancer cells morphology

Using visible light microscopy, no significant differences in the surface morphology of white blood cells could be detected in cells from chronic leukemia patients and healthy subjects. However, observations of both cells under AFM revealed that a large number of needle-like structures appeared on the surface of leukocytes in leukemia patients, and that cell surface roughness was also significantly higher than that of normal white blood cells [31]. This indicated that the resolution of the AFM is significantly higher than that of the optical microscope, and is capable of distinguishing between differences in the ultrastructure of the cells. In addition to observations of tumor cell lines, AFM can also scan patient’s tumor tissue or cells for imaging. Hanekar et al. explored the relationship between SMAR1 expression and cell surface roughness under AFM in different grades of human breast cancer tissues [32]. This study illustrates that it is possible to observe the ultrastructure of the membrane surface of different types of tumor, and to analyze their common characteristics and differences. AFM can also highlight the significant differences in cell membrane morphology between normal cells and tumor cells, and can determine whether the cells are malignant or not, compared with tumor cells from the same source. This provides a reliable auxiliary basis for clinical pathological and differential diagnoses.

**Fig. 4:**
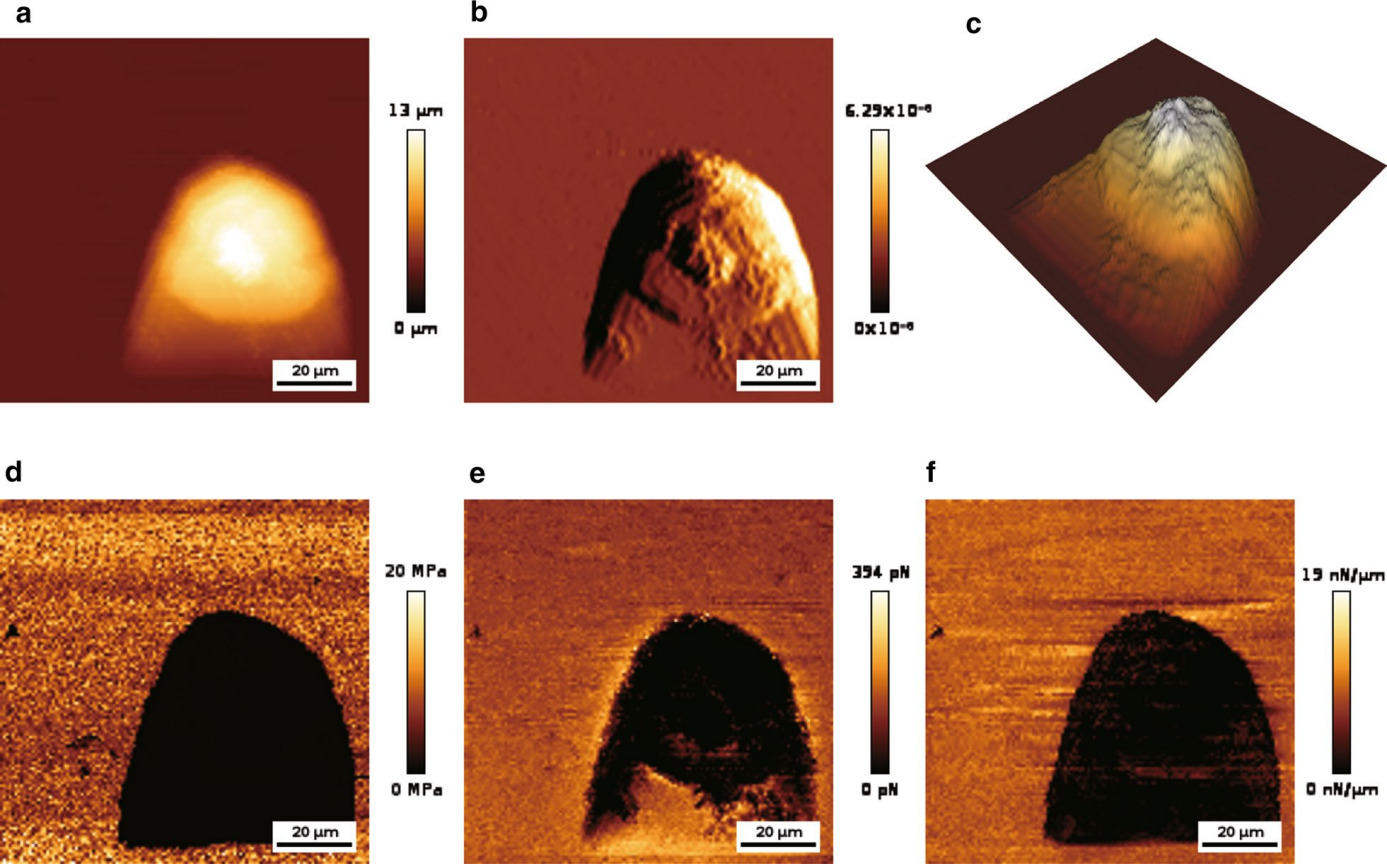
Imaging of cancer cell morphology and measuring the mechanical properties of cancer cells by AFM. **a** Representative AFM height image. **b** Representative AFM deflection image. **c** 3D distribution of cell height. The force curve is used to calculate mechanical properties. **d** Image representing Young’s modulus distribution. **e** Image representing adhesion force distribution. **f** Image representing stiffness distribution. Parameters are displayed as colors. The results of morphology observation and mechanical properties measurement of the cell were from our study group

In cancer cells, the expression of certain genes may be either up-or down-regulated. For example, the CDX2 gene is less active in most patients with colorectal cancer [33, 34]. Overexpression of CDX2 can inhibit the metastasis of this cancer, while further inhibiting its expression promotes metastasis [35, 36]. It has been shown that CDX2 has tumor suppressing activity [37–39]. AFM detection showed that the stiffness of colon cancer cells increased with higher expression of CDX2, and that cell variability decreased, which weakened the ability of the cells to transfer to the extracellular matrix and capillaries [40]. Vascular endothelial growth factor D (VEGF-D) and vascular endothelial growth factor C (VEGF-C) are thought to be involved in the formation of lymphatic vessels and blood vessels [41, 42]. After transfecting a recombinant plasmid containing VEGF-D into lung cancer cells, AFM detection showed that irregular micro-spikes and nanoclusters appeared on the cell surfaces, and that cells became more rigid. Using AFM to observe the surface morphology of lung cancer cell PC9, the morphology image of cancer cells with high resolution could be obtained (Fig. 4a–c). The above studies demonstrate that AFM can be used to detect changes in the morphology of cancer cells caused by cancer-associated genes, providing an additional detection method for phenotypic changes which is complementary to traditional investigative methods.

## Mechanical properties of cancer cells

The mechanical strength of cells plays an important role in the homeostasis of tissues, cell growth, division, migration, and epithelial-mesenchymal transition [43]. Numerous studies have shown that differences exist in the stiffness of normal and cancerous cells, and between primary and metastatic cancer cells. Cross et al. detected the cells of patients with suspected metastatic adenocarcinoma (lung, breast, and pancreatic cancer) using AFM. Studies have indicated that, even if benign and malignant cells are similar in shape and therefore difficult to distinguish by visual inspection, they may still be identified by mechanical analysis [44, 45]. In addition, the Cross group also reported biomechanical differences between human mesothelial tumor cells and normal cells [46]. Other researchers have found that human bladder cancer cells have lower hardness than normal epithelial cells [47], and that human breast cancer MCF-7 cells are softer than MCF-10 cells [48]. These studies showed that it is possible to distinguish between cancer cells and normal cells, and between original and metastatic cancer cells, by detecting their mechanical properties using AFM. This provides a novel auxiliary means of clinical cancer diagnosis. The mechanical properties of lung cancer cell PC-9, including Young’s modulus, adhesion, and stiffness, have also been quantitatively measured by AFM (Fig. 4d–f). This showed that AFM can not only detect the morphology of cancer cells, but also perform deeper mechanical properties and cell structure analysis on cancer cells.

The invasion and metastasis of tumor cells has an important relationship with the microenvironment. AFM measurement of extracellular matrix (ECM) micromechanics provides important insights into disease-induced tissue stiffness alterations [49]. It was found that normal liver tissue, liver fibrosis tissue, and liver cirrhosis were significantly different in terms of hardness when examined by AFM. There was no significant difference in hardness between liver cirrhosis tissue and hepatocellular carcinoma tissue [50]. This suggested that the hardness of the liver is increased during carcinogenesis, which may progress to liver cancer after cirrhosis. In addition, a series of studies have shown that cancer cells are more rigid than normal cells [51]. These findings disprove the idea that cancer cells tend to be softer than normal cells, and indicate that hardness depends on the needs of the cancer cells themselves during the process of carcinogenesis.

## The study of tumor-associated molecules and subcellular structures

Circulating cell-free DNA (ccfDNA) is an important biomarker for the diagnosis and treatment of cancer [52], and can be indicative of changes in the genotype and phenotype of primary tumors [53]. It has been found that more than 80% of the ccfDNA fragments in the plasma of cancer patients are under 145 bp in size, and that their size is always less than 300 bp [54]. Reduced fragmentation of ccfDNA is associated with increased overall survival. After chemotherapy, plasma ccfDNA fragmentation in normal patients is greatly reduced, while patients with KRAS mutations have a greater degree of radiotherapy resistance [55]. This demonstrated that AFM can be used to detect the ccfDNA fragmentation index in cancer patients, and that this may be used as a potential biomarker for cancer treatment [56–59].

miRNAs play an important role in controlling various cellular processes, and their expression levels can change with disease [60–65]. Northern blotting is a standard method for miRNA quantification, but requires a large amount of RNA for analysis and is insensitive to low abundance RNA [66, 67]. qRT-PCR is also used to quantitate miRNAs, but the length of miRNAs and primers will, to some extent, hinder their direct detection, which may also be affected by platform differences [68]. Neither of these techniques can be used to perform in situ analysis of miRNAs, or to provide information concerning their subcellular localization. Although nanopore sensors and the single-molecule fluorescence microscopes can be used for single miRNA detection, their sensitivity is not strong [69–72]. While enzyme-assisted fluorescence [73], nanoparticle [74], and in situ enrichment (Toehold-initiated rolling circle amplification) [75] techniques can observe single-molecule miRNA, their spatial resolution is poor. AFM, however, is perfect for accurately quantifying miRNA content at the single cell level, allowing for direct and high sensitivity in situ quantification [76]. AFM cannot only quantitate intracellular miRNAs, but can also provide accurate information on their subcellular localization [77]. Therefore, cancer-associated miRNA detection can be performed in situ using AFM, which further extends the diagnostic methods available for cancer [78, 79]. In addition, there are miRNAs in the exosomes secreted by tumor cells which are considered to be related to the development of tumors. These constitute a variety of tumor markers for breast cancer [80–82], colon cancer [83, 84], lung cancer [85], thyroid cancer [86], etc. AFM can be used for imaging analysis of exosomes with different separation modes, and can analyze the effects of different separation methods on the size of exosomes [87]. It can therefore be used to detect cancer diagnosis markers including ccfDNA, miRNA, and exosomes, strongly supplementing existing cancer detection and diagnosis methods.

In addition, AFM can be used to observe the internal structure of cancer cells. All signaling pathways for cancer cell metastasis go through the nuclear pore complex (NPC), and small molecules can freely pass through NPCs [88]. However, molecules larger than 40 kDa must be combined with FG-Nups to pass through the nuclear pore complex [88]. Using high-speed AFM (HS-AFM), researchers were able to directly observe nano-scale changes in the channels of the nuclear pores in dying colon cancer cells (CRC), and found that these changes were mainly in FG-Nups [89]. This suggested that HS-AFM can measure high speed dynamic changes and disordered intracellular systems, and may be used as a cell endoscope to study other organelles in cancer cells. These results showed that AFM can not only measure the morphology and mechanical properties of cells, but can also be used to locate and analyze corresponding molecular and subcellular structures in cancer.

## The study of the interaction between molecules

**Fig. 5:**
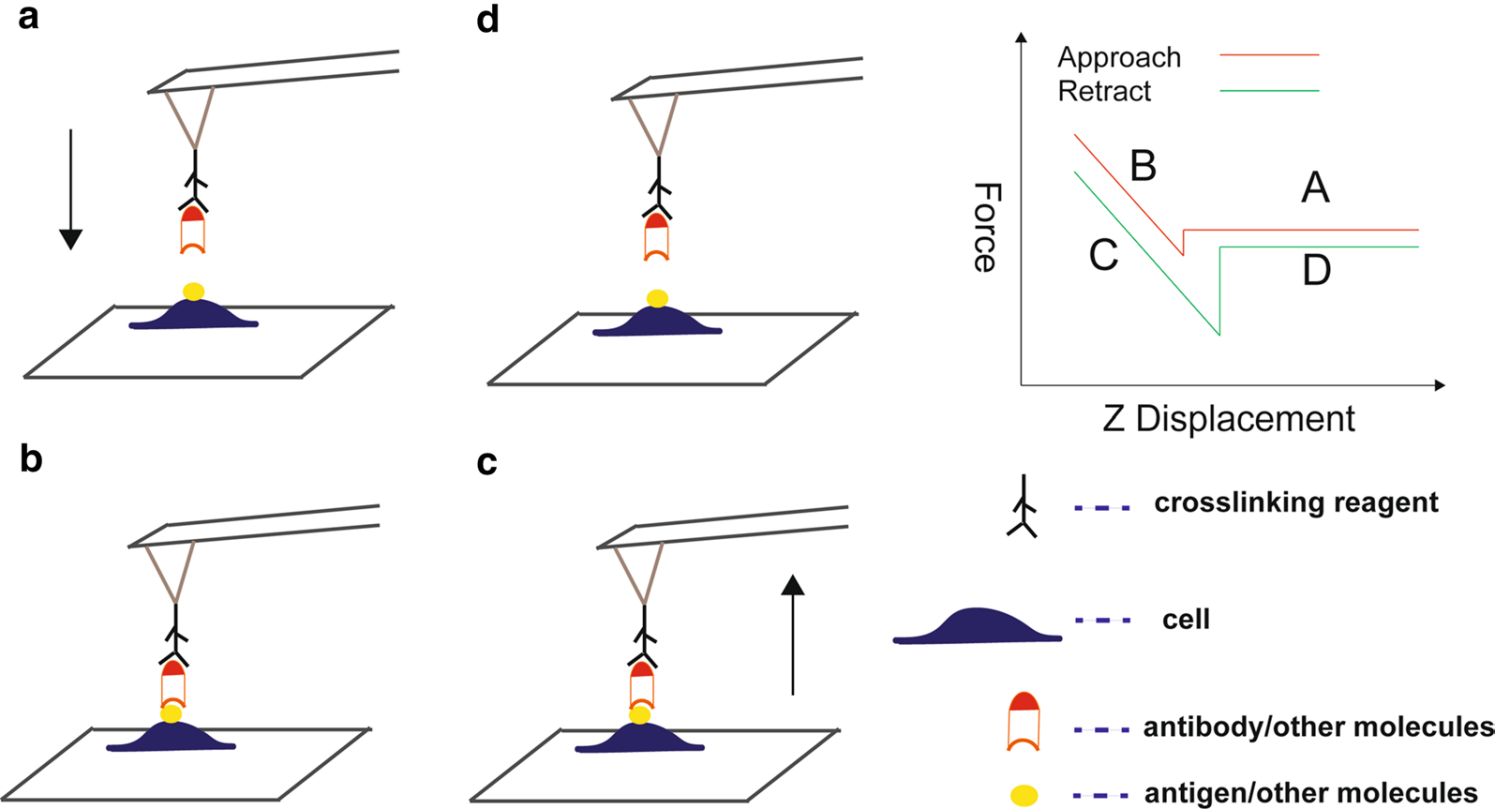
Schematic diagram of AFM-based single-molecule force spectroscopy. **a** Individual antibodies or other molecules immobilized on a functionalized tip are positioned above a cell adhering to a substrate. **b** Sample and tip molecules are then brought into contact for a defined contact time and with a preset contact force. **c** The molecules are subsequently separated, and the maximal separation force and the detachment work can be determined using a simultaneously recorded force–distance curve. **d** The cantilever is retracted until the tip and sample molecules separate

For cells, AFM can not only provide morphological information on their length, width, and height, but can also be used to study fine structures such as receptors, lipid rafts, and various pathways on membranes. AFM allows researchers to study the activity of unique molecules on individual cells under physiological conditions [90]. By attaching specific molecules (such as ligands and antibodies) to AFM probes, they can locate and manipulate interacting proteins (such as receptors and antigens), using a method called single-molecule force spectroscopy (SMFs) [91, 92] (Fig. 5). This is a powerful supplement to traditional methods to detect the interaction between single molecules [93]. For example, Rituximab is a monoclonal drug for the treatment of leukemia, which is connected to the AFM probe surface [94] to measure the distribution and binding of CD20 and FCR in tumor and NK cells, respectively, and it was found that the binding force of CD20-rituximab is significantly greater than that of FCR-rituximab, the frequency distribution of CD20 in tumor cells was significantly higher than that of FCR on NK cells. The above research suggested that the study of interactions between molecules is feasible by AFM, which can also be used to study the interactions between cancer-related molecules.

Curcumin is a natural polyphenol complex, and a large number of studies have shown that it has an inhibitory effect on many cell lines [95–97], but has no toxic effect on normal cells [98–100]. The CD44 antibody was connected to the AFM probe surface to measure CD44 distribution of the cell surface and the binding force between CD44 and the antibody. Researchers found that the binding force between the CD44 antibody and surface CD44 in human hepatoma cells treated by curcumin was decreased, and the expression of CD44 on cell surfaces was also decreased in unit area [101]. This finding is similar to the results detected by the flow cytometry [101]. These studies help to explain the relationship between the distribution of CD44 and the apoptosis of cancer cells, as suggest the possibility of tumor therapy by blocking the CD44 molecule on the tumor cell with CD44 antibody. Results of this type show that AFM has good application value in the study of tumor-related molecules.

AFM has many advantages in detecting the interaction between molecules. Compared with traditional methods such as surface plasmon resonance [102], radioimmunoassay [103], and fluorescence resonance energy transfer [104], SMFs can be used to directly measure molecular interactions on the surface of living cells, but in relatively shorter time and at lower cost. The information obtained from SMFs analysis in conjunction with clinical data can help researchers discover biomarkers for efficient tumor prediction and diagnosis.

## Evaluation and development of antitumor drugs

It is of potential value to estimate the effect of anticancer drugs by regulating changes in the morphological and mechanical properties of cancer cells [105–107]. It is generally believed that decreased adhesion of cancer cells is the first step in metastasis. Disulfiram (DSF) is a commonly used clinical hangover drug [108]. However, studies have shown that the DSF-Cu complex has an anti-tumor effect [109]. The stiffness and adhesion of nasopharyngeal carcinoma cells treated with DSF-Cu have been shown to be increased [110], which suggested that DSF-Cu reduces the deformability and enhances the adhesion of these cells. DSF-Cu may, therefore, inhibit the malignant growth and metastasis of nasopharyngeal carcinoma cells.

Changes in the deformability and adhesion of cancer cells may be due to major changes in the morphology and structure of the cell surface following drug treatment. After treatment of colon cancer cells with AEE788 and Celecoxib, their cell surfaces became rough and severely contracted, with pseudopodia and filopodia disappearing completely [111]. The morphology and ultrastructure of Hela and HepG2 cells were also changed after being treated by colchicine and cytidine, and the degree of damage to cancer cells varied with the concentration of the drugs, and the timing of their administration [112]. These findings suggested that the surface morphology of cells can change dramatically after stimulation by drugs or bioactive compounds. In addition, the efficacy of drugs can be judged by their effects on cell surface roughness and hardness. Prostate cancer is a malignant tumor that occurs in the prostate gland of men. Ren et al. evaluated the effects of eight anticancer drugs against this cancer using AFM [113]. They found that the Young’s modulus of PC-3 cells increased significantly after treatment with eight drugs, and the mechanism of action of drugs on the mechanical properties of PC-3 cells was different. These studies suggested that AFM can be effectively used in the screening and evaluation of anticancer drugs.

The development of new anti-cancer drugs is essential for the treatment of cancer. Most of the anti-tumor drugs are cytotoxic substances. Due to their poor specificity, they can also cause damage to human normal cells, and may cause strong adverse reactions while providing only low anti-cancer efficiency. Traditional liposomes and nanomaterials have been shown to have good biocompatibility, sustained release, and targeting after being modified and improved, and are the most attractive drug carriers. In evaluating the physical properties and stability of liposomes, AFM is one of the most effective detection technologies [114]. Lapatinib is a dual inhibitor that is used to treat advanced breast cancer and some other cancers [115–117]. Lapatinib is poorly soluble in water, but after being encapsulated in lipoproteins to form nanoparticles (LTNPs), its water solubility increases from 7 μg/mL to 10 mg/mL or more. AFM measurements showed that LTNPs are spherical particles, and that the height of the particles is not positively correlated with the diameter, as the diameter is about 60 nm and the height is less than 3 nm [118].

Carbon nanomaterials are widely used in biomedical devices and biosensors, including carbon nanoparticles (CNP), carbon nanotubes (CNT), and graphene, etc. [119–121]. One study found that carbon nanoparticles (CB) which were covered using BSA and surface-coupled to methotrexate (methotrexate) to form complexes (CBM) were better than methotrexate alone for inhibiting tumor cells [122]. The sizes of CNP, CB, and CBM were found to range between 16–27, 35–44, and 51–66 nm, respectively, using AFM. Surface morphological changes were found on functionalized CB and CBM, which was mainly reflected in the significant change in roughness [122]. Similar conformational changes were also found when BSA was bound to CNP [123, 124]. This indicated that CNP could be used as a nano-carrier to connect anticancer drugs with their corresponding target cells. The surface structure of these nanoparticles or nanocomposites can be quickly determined by AFM [125], allowing for the possible function and work scope of the nano-composites to be predicted.

AFM can assist in the evaluation, screening, and development of anti-cancer drugs. Therapeutic methods that can cause changes in the morphology and mechanical properties of cancer cells can be detected by AFM, which provides an aid for the diagnosis and treatment of cancer.

## AFM in combination with other technologies

The distal metastasis of cancer is the cause of death in most cancer patients. Understand the differences between primary cancer cells and metastatic cancer cells is therefore critical for the diagnosis and treatment of cancer. At present, it is known that the original cancer cells and metastatic cells have different genetic maps [126, 127], which can be used in clinical diagnosis. It has been shown that Raman spectroscopy can successfully distinguish between cancer and healthy cells, primitive and metastatic cancer cells, and cancerous and healthy tissues [128–131]. The prominent advantage of Raman spectroscopy is that it is possible to know the chemical composition of the substance [132], which is impossible for AFM. However, if AFM and Raman spectroscopy are used together [133], it is possible to not only know the substance content of the cell surface, but to simultaneously determine the substance’s chemical composition, thereby enlarging the scope for the use of AFM.

AFM may be combined with a confocal laser scanning microscope (CLSM) to study the mechanical properties of individual cancer cells [134]. The combination of AFM and CLSM directly correlates indentation points with film imaging of subcellular structures [135]. In the early stage of cancer development, the mechanical properties and morphology of cells change. Researchers can detect changes in the elasticity of cancer cells using AFM and elucidate its relationship to malignancy in the early transformation process. Alpha-enolase (ENO1) is a multifunctional protein [136, 137] that is not only one of the enzymes involved in glycolysis, but also a fibrinogen receptor that promotes cancer metastasis [138–141]. Combined use of AFM and CLSM revealed that ENO1 gene silencing made pancreatic cancer cells coarser, damaging the adhesion between cancer cells, and between cancer cells and stroma, resulting in the invasion and metastasis of pancreatic cancer cells [142].

Cervical cancer is the second most common cancer in women [143]. Accurate and early diagnosis can reduce costs, greatly benefiting patients. AFM’s high resolution imaging of suspected malignant cells can provide more information than light microscopy, targeting cells for accurate diagnosis [144, 145]. It also provides advantages over scanning electron microscopy (SEM) imaging, allowing for high-resolution 3D imaging, with faster sample preparation, and determining the unique mechanical properties of cells. However, there are limitations to the imaging of cervical cancer cells using either AFM or SEM alone. Multiple forms of detection are more accurate than those provided by a single format test. The imaging diagnosis of cervical carcinoma cells with AFM and SEM has the advantages of fast detection, easy operation, low cost, and so on [146]. SEM and AFM are currently the most common means of surface analysis. However, with AFM, work area selection is very blind, and the work area itself is very limited. Scanning can only be done on the micrometer scale, and it is very difficult to scan larger sample surfaces. Utilizing the large-scale search capabilities of SEM to find and target features or areas of interest that need to be studied by AFM can greatly improve the efficiency of analyses. This system can also be used to detect and diagnose other cancers.

The World Health Organization classifies astrocytomas based on the microscopic appearance of tumors [147]. However, the details of the complex morphology vary according to the specific tumor and patient, and the final classification has to be determined by an experienced pathologist. Therefore, in order to make the classification more accurate, more objective criteria need to be determined. The combined use of AFM imaging and Data Mining Techniques to classify brain tumors is a recently-developed method, achieving 94.74% classification accuracy in distinguishing between type II, III, and IV tumors [148]. Patients with stage II tumors can quickly be diagnosed using this method, reducing their risk of cancer metastasis.

AFM imaging and mechanical measurement techniques have been widely used in the biomedical field, and allows for the determination of cell structure and function from level of single molecules to that of individual cells. These methods can be used to differentiate between cancer and normal cells at the single-cell level, and enables visual drug research. No technology lacks drawbacks, and AFM technology does have limitations, but its use in combination with other complimentary technologies is a developing trend and is expanding the use of AFM.

## Conclusion and prospects

After nearly 30 years of development, AFM has played an increasingly important role in cell imaging and the study of the mechanical properties of cells. From initial imaging modes (such as contact and tapping imaging modes) and force curve-based mechanical measurement modes to today’s high-speed imaging mode (such as fast-scan mode) [149] and high-resolution mechanical measurements modes (such as peak force mode) [150, 151] make AFM increasingly important in the study of cell physicochemical properties. The mechanical properties of cells can be used as a new diagnostic physical biomarker to supplement traditional tissue detection [152]. However, there are still many problems to be solved before the application of AFM technology can be fully realized in practice (for example, in the diagnosis of cancer).

The usage efficiency and the temporal resolution should be improved. At present, AFM measurement of cells mainly depends on manual operation, including manual control of the probes to target cells, manual parameter setting to obtain cell force curves, and manual offline processing of experimental data. This leads to very low experimental efficiency, and it takes several minutes to take a measurement of a cell [8]. In particular, in order to obtain statistically significant conclusions, many cells need to be measured, which results in a large workload, and limits the practical application of AFM at the single cell level. Therefore, improving AFM’s level of automation will help improve its measurement efficiency. In addition, the response time of the cell to the environment is approximately 1 ms, which is significantly shorter than AFM’s mechanical mapping time (about 10 min) [153]. This has made it difficult to monitor changes in the mechanical properties of cells in real time. Although HS-AFM has been commercialized [154], with available systems having imaging times of less than 100 ms [155], and is suitable for imaging rigid and flat small-size samples, such as substrate-bound molecules [156] and microbial cell walls [157], increasing the speed of AFM detection will be of use in real-time studies of changes in the dynamic mechanical properties of cells [158, 159]. High-speed AFM can also be used for imaging of mammalian cells [160]. The imaging time is, on average, approximately 5 s, far larger than the response time of cells to external stimuli.

The standardization of AFM measurements is another requirement. Young’s modulus is a value commonly reported when AFM is used to measure the mechanical properties of cell, but is dependent on the conditions of the experiment, including the environment (temperature, substrate, culture medium) [161], instrument parameters (loading rate, indentation depth) [162], cell (cell position constrained, cell state) [163], data analysis (e.g., pattern selection, contact point determination) [164], and so on. The results of different studies can only be compared when the conditions are in full agreement, and it is difficult for researchers to maintain identical experimental conditions. Therefore, in order to make the measurement results of different research groups comparable, the measurement process needs to be standardized, as does sample treatment.

For clinical and practical applications, multiple clinical cases are needed to verify the reliability of mechanical property detection. Sample preparation and AFM measurements need to be standardized, and the relative values characteristic of cancer cells and normal cells need to be determined. Some studies directly measure the mechanical properties of the original cancer tissue, which allows for detection of the different stages of tumor metastasis [165, 166]. However, tumor tissue includes cancerous cells, normal cells, blood vessels, and extracellular matrix [167], which means that such measurements describe the mechanical properties of the local tissue, and not of isolated cancer cells. The most difficult part of the measurement is to determine the contribution of each component to the mechanical properties of cancerous tissue. Studies have shown that other non-tumor cells in tumor tissues also affect the development and metastasis of tumors [168]. In addition, tumor tissue is highly heterogeneous, with differences manifesting in different tumor tissues as well as in different parts of the same tissue [169]. Therefore, tumor tissues in different locations may have different mechanical properties, and different parts of the tissue need to be extracted for comparative detection.

In recent years, many AFM models have been developed [170–172]. Together with advances in complementary techniques (Table 2), this will allow AFM to address outstanding questions in biology in the coming decades. These have made the AFM easier to apply to biological systems, which has resulted in more information on these systems being generated.

**Table 2:** Comparison of high-resolution imaging techniques in molecular and cell biology [171]

Technique/feature	Atomic force microscopy	Super-resolution microscopy (STED, PALM, STORM)	Transmission electron microscopy	Scanning election microscopy
Resolution	≤ 1 nm–50 nm	20–50 nm	0.2–10 nm	2–10 nm
Sample preparation and environment	Sample on support; physiological (buffer solution, temperature, CO_2_)	Fluorescence labelling; physiological (buffer solution, temperature, CO_2_)	Sample on grid; dehydrated (negative stain); vitrified (cryo-electron microscopy)	Freeze/critical point drying and metal shadowing
Artefacts	Tip, force, scanning	Bleaching, toxicity	Dehydration, ice crystal formation, beam damage	Dehydration, metal shadowing, beam damage
Advantages	Imaging under native conditions; no staining, labelling, or fixation necessary; high signal-to-noise ratio; assessment of multiple physical, chemical, and biological parameters	Access to three-dimensional cellular structures; high spatiotemporal resolution; monitoring biomolecular processes in life cells	Solves atomic structures of proteins; conformational snapshot of proteins and complexes; molecular resolution of structures within the cell	Imaging surfaces of tissues, cells, and interfaces as nanometer-scale resolution
Limitation	Restricted to surfaces	Imaging restricted to fluorescence labels	No life processes	No life processes

*STED* stimulated emission depletion, *PALM* photo activated localization microscopy, *STORM* stochastic optical reconstruction microscopy

Currently, the sensitivity and temperature stability (drift) of AFM limits its accuracy in describing biological systems. The recently introduced ultra-stable AFM can provide the accuracy of sub-pico force while providing high stability (< 0.03 Å) at very low lateral drift (~ 5 pm min) [173, 174]. We predict that with the continuous development and improvement of AFM technology, it will play an increasingly important role in cancer research and diagnosis.

## Abbreviations

AFM: atomic force microscopy
CB: carbon nanoparticles coated in BSA
CBM: carbon nanoparticles coupled to methotrexate
ccfDNA: circulating cell-free DNA
CLSM: confocal laser scanning microscope
CNP: carbon nanoparticles
CRC: colon cancer cells
CNT: carbon nanotubes
DSF: disulfiram
HS-AFM: high-speed AFM
LTFNs: lipoproteins to form nanoparticles
NPC: nuclear pore complex
SMFs: single-molecule force spectroscopy
VEGF-D: vascular endothelial growth factor D
VEGF-C: vascular endothelial growth factor C
ECM: extracellular matrix
